# Awareness of diabetic foot disease amongst patients with type 2 diabetes mellitus attending the chronic outpatients department at a regional hospital in Durban, South Africa

**DOI:** 10.4102/phcfm.v8i1.1170

**Published:** 2016-11-17

**Authors:** Thea T. Goie, Mergan Naidoo

**Affiliations:** 1 Discipline of Family Medicine, School of Nursing and Public Health, University of KwaZulu-Natal, Durban, South Africa

## Abstract

**Background:**

Diabetic foot disease (DFD) is a major challenge for the healthcare system, with enormous economic consequences for people living with diabetes, their families, and society, affecting both quality of life and quality of care. The study aim was to assess the level of awareness of DFD amongst patients with type 2 diabetes mellitus (T2DM).

**Methods:**

An observational descriptive cross-sectional study was conducted at the chronic outpatients department of a regional hospital in Durban, South Africa.

**Results:**

Two hundred participants with T2DM participated in the study. Ninety-one per cent of participants were either overweight or obese. Ninety-two per cent of participants had concomitant hypertension (57.5%), dyslipidaemia (26.7%) and eye disease (7.2%). Seventy-six per cent reported altered sensation in their lower limbs, and 90% reported having no previous DFD education. Only 22.2% of participants reported having examined their feet, but only when they experienced a problem. Participants achieved mediocre scores for knowledge (mean 4.45, standard deviation (s.d.) 2.201, confidence interval (CI) 4.2–4.7) and practice (mean 11.09, s.d. 2.233, CI 10.8–11.5) on diabetic foot care (DFC). Those who had a higher level of education and who were less than 65 years old had a significantly better score for previous foot care education (*p* <0.05).

**Conclusion:**

The study demonstrated that awareness of DFD was suboptimal, based on current DFC guidelines. To minimise the burden of DFD, improved screening and prevention programmes as well as patient education should be provided to T2DM patients, whilst maintaining an aggressive approach to risk factor modifications, footwear and identifying the at-risk foot.

## Introduction

More than 350 million people worldwide were known to have diabetes mellitus (DM) in 2013, and an estimated 592 million are expected to have the disease by 2035. Most of these people are between 40 and 59 years of age, and live in low- and middle-income countries (LMICs).^1,2^ It is also estimated that 50% of people with DM are undiagnosed. The World Health Organization (WHO) estimates that DM will be the seventh leading cause of death in the next 15 years.^1^

The five sub-Saharan African countries with the highest rate of type 2 DM (T2DM) are Nigeria, South Africa, Ethiopia, the Congo and Tanzania.^2,3^ Owing to the ‘westernisation’ of rural African communities, the prevalence rate of DM has increased amongst persons of indigenous descent.^3,4^ In South Africa (SA), the prevalence of DM amongst the black population is second only to the prevalence of South Africans of Indian descent.^5^

One of the most common and distressing complications that affects diabetic patients is diabetic foot disease (DFD).^6,7^ DFD comprises a constellation of vascular and neurological pathologic changes that are the direct result of DM, causing local tissue destruction by sensory neuropathy and compromise of the vascular system of the affected lower extremities in diabetic sufferers.^1,8^ These contributory factors co-exist in more than 10% of patients at the time of diagnosis of T2DM.^7,8^ DFD is a major challenge for the healthcare system in both high-income countries and LMICs, with substantial economic consequences for the patients, their families, and society.^6,7^ DFD accounts for 20% of all hospitalisations of T2DM patients in sub-Saharan Africa, and frequently leads to chronic disabilities, loss of income, lower limb amputation or death.^7,9,10,11^ It is estimated that one in every five persons with DM (type 1 and type 2) has a 15% probability of developing a foot infection in a year, and 5% of DM patients with DFD will eventually undergo amputation.^12,13,14^ Despite various interventions, DFD remains a common and significant clinical problem affecting quality of life and quality of care that disrupts patients’ psychosocial and physical state and has a negative impact on their overall perception of the disease.^15,16,17^ DFD leads to physical limitation and functional disability.^15,16,17,18^

Considering that the patient is the primary foot carer, it is essential that to reduce the incidence of foot disease amongst patients with T2DM, they have a good awareness of the risk factors that could predispose or worsen DFD, as well as awareness of good foot care practice.^6,9,13,19,20,21^ Commonly, DFD develops in areas of the foot exposed to continuous pressure, friction and repetitive trauma. Harmful footwear such as those with unergonomic interiors, high heels and narrow foreparts, is one of the major precipitating causes implicated in the progression to DFD and amputation. Inappropriate shoes are sometimes considered ‘enemies of the oppressed foot’.^22,23^ Evidence shows that knowledge is associated with better attitudes and practices of foot care, and should consequently bring clinical benefit – although this is not always the case, as the quality of care offered at primary healthcare (PHC) level in our settings is often unsatisfactory.^24,25^ More studies need to be performed to evaluate whether clinical benefit may arise from education-targeted community programmes in comparison with the usual care provided.^25^ Awareness of good foot care is essential amongst T2DM patients and health care providers to reduce the incidence of foot disease, and this would involve:

- preventing and managing local trauma and/or infection
- dealing with foot deformities
- managing abnormal pressure points
- improving poor glycaemic control
- managing pre-existing vascular damage and/or peripheral neuropathy
- managing associated cardiovascular diseases
- improving awareness and self-practice of foot care.^25,26^

Awareness involves the ability to know and understand those factors that will further develop beliefs, and dictates attitudes and practices toward responsibility, improvement and success. The aim of the present study was to assess the level of awareness of DFD amongst patients with T2DM who attend the chronic outpatients department (OPD) at a regional hospital in Durban, SA. The specific objectives included briefly assessing the clinical profile of T2DM patients and evaluating their knowledge, attitudes and practices concerning DFD.

## Methods

The study was an observational descriptive cross-sectional study that was conducted at a regional hospital in the city of Durban in KwaZulu-Natal, SA, during October 2014. The hospital is a 1200-bed institution that serves a large catchment area consisting of urban and rural populations. It provides district and regional levels of care to patients, with an average of 43 000 patients visiting OPD monthly.

The hospital’s OPD for chronic patients is run by the Department of Family Medicine, and diabetic clinics are on Mondays, Thursdays and Fridays. The chronic OPD provides ambulatory care to approximately 700 diabetic patients monthly. A sample size of 280 which represents approximately 36% of the monthly patient load was considered appropriate for the study, after consultation with the biostatistician. The inclusion criteria were T2DM patients who were ≥18 years, had been on treatment for ≥12 months, and attended the diabetic clinic at the chronic OPD at this hospital.

Patients who did not consent to participate in the study, those who were cognitively impaired and those who had debilitating mental illnesses were excluded from the study. A systematic randomised sampling method was used to select participants. Every third patient meeting the inclusion criteria was asked to participate in the study. Measures were in place to ensure that participants did not participate in the study more than once.

Validated questionnaires used to assess DFC in previous studies were adapted for the present study.^27,28,29,30,31^ The adapted questionnaire consisted of options with sections that covered socio-demographic and clinical profiles of patients, and knowledge, attitudes and practice variables. A pilot study of 10 patients allowed the modified questionnaire to be used. The questionnaire was translated into the local language isiZulu for patients who could not speak/read English, and back-translated into English to ensure accurate translation. A research assistant who was proficient in isiZulu was trained and worked with the principal investigator throughout the process of data collection. Signed written informed consent was obtained from each participant. The questionnaire and an information sheet were distributed by hand to each participant. All questionnaires were filled out anonymously to protect confidentiality of participants. Minimal information was collected from participants’ medical records. Ethics approval to conduct the study was granted by the Biomedical Research Ethics Committee (BREC) of the University of KwaZulu-Natal (reference number 290/14) and the provincial Department of Health. Data were captured on an Excel spreadsheet and analysed using IBM SPSS version 23 by the biostatistician. Linear correlation (Pearson coefficient), *p* values and confidence intervals were calculated to ascertain the statistical significance of any correlation found. The level of significance was set at *p* < 0.05.

## Results

**FIGURE 1:**
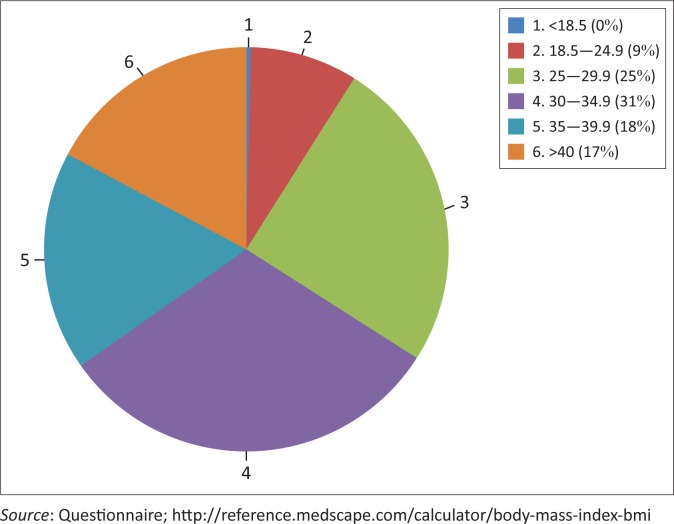
Body mass index frequencies.

Two hundred and ninety-nine participants gave informed consent and received the questionnaire. Nineteen participants dropped out whilst filling in their answers, some saying that they had to rush elsewhere, and others for no particular reasons. A total of 280 participants with T2DM participated in the study, of whom 201 (71.8%) were female and 79 (28.2%) were male. Their mean age was 59±9.28 years. Most participants were black (89.6%). There were 27 (9.6%) Indian participants, one white participant and one of coloured origin (0.4% each). One hundred and sixty participants were married. Ten participants (3.6%) had tertiary education, of whom 60% were female. Fifty-four (68%) of the male subjects had no formal education. Almost 59% of participants (*n* = 164) received between R1000 and R1999 monthly, comprising government social grants, and 29% of participants (*n* = 81) earned <R500. Ninety-one per cent of participants were either overweight or obese (Figure 1).

Eighty-four (30%) participants had been diagnosed with T2DM in the past 5 years, and 21.4% of participants possessed a glucometer. Most (95%) participants only visited the diabetic clinic for their treatment, and 91.8% visited the clinic monthly. Ninety-five per cent of participants had concomitant existing medical conditions. The three most common comorbidities were hypertension (57.5%), dyslipidaemia (26.7%) and eye disease (7.2%). Concomitant peripheral neuropathy was found in 3% of participants. All participants were on medication for T2DM, with 12 participants stating that they were not taking their medication regularly (*n* = 5) or had defaulted on their treatment (*n* = 7). Seventy per cent of participants practised one or more forms of exercise, of whom 65.7% (*n* = 130) exercised for 30 minutes a day or 150 minutes per week. Others (13%; *n* = 27) exercised <30 minutes per day or had an irregular exercise schedule (*n* = 26). The most practised form of exercise was walking (49.8%), followed by gardening (36.3%) and jogging (8.50%). Eighty-seven per cent of participants claimed to be on a low-fat no-sugar diet, with 66.8% observing this diet sometimes and 33.2% claiming to be always compliant with this dietary regime. Ninety-seven (34.6%) patients monitored their body weight regularly.

Table 1 represents the distribution of patients regarding previous and current DFD. Some patients had one or more types of DFD.

**TABLE 1:** Number of participants with previous and current diabetic foot disease.

	Yes	
	
Symptoms	*n*	%
Previous ulcer on feet	26	9.3
Previous amputation	11	4.0
Current ulcer on feet	10	3.6
Current blood/discharge on feet	6	2.1
Current calluses	37	13.2
Current numbness and tightness in lower limbs	214	76.4

**FIGURE 2:**
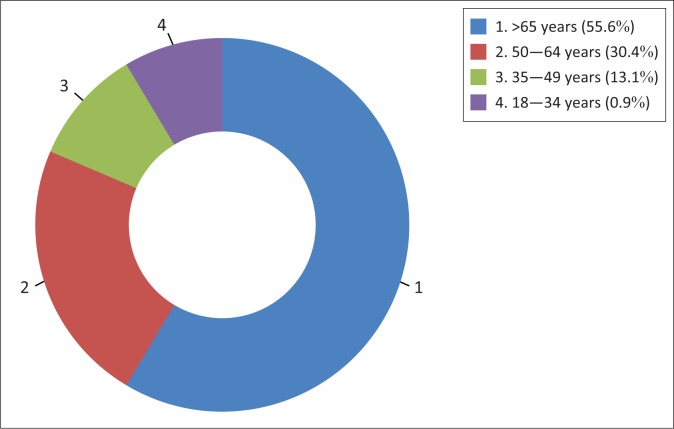
Distribution of participants with current numbness and tightness in lower limbs per age ranges.

Most (76%) participants reported having altered sensation (i.e. tightness and numbness) in their lower limbs at the time of the interview. These participants were mostly aged between 50 and 64 years (55.6%) with an additional 13% between the ages of 35 and 49 years who also reported having these symptoms (Figure 2). Seventy-one per cent of participants with altered sensation were diagnosed with T2DM for 5 years and more.

Table 2 presents the level of knowledge and attitudes of DFC and previous diabetic foot education. Table 3 presents the level of practice of DFC.

**TABLE 2:** Knowledge and attitudes about diabetic foot care and previous diabetic foot education.

	Yes		No	
		
Knowledge and attitudes	*n*	%	*n*	%
DM patients may develop reduced blood flow in their feet.	147	52.5	133	47.5
DM patients may develop lack of sensation in their feet.	180	64.3	100	35.7
DM patients may develop foot ulcers.	167	59.6	113	40.4
DM patients may develop gangrene.	204	72.9	76	27.1
Are you aware that smoking can reduce blood flow in your feet?	175	62.5	105	37.5
Do you know that with loss of sensation in your foot, you are more prone to foot ulcers?	107	38.2	173	61.8
Do you know that with reduced blood flow in your foot, you are more prone to foot ulcers?	109	38.9	171	61.1
Do you know that if you have foot infection, you will develop foot ulcers?	160	57.1	120	42.9
DM patients should take responsibility for self-foot examina-tion.	234	83.6	46	16.4
You can lead a normal life if you take appropriate measures to control your DM.	248	88.6	32	11.4
Diet is important in the control of DM.	265	95	14	5
Have you ever attended a class on how to care for your feet?	3	1.1	277	98.9
Have you ever received education about foot care from the nurse?	11	3.9	269	96.1
Have you ever received education about foot care from the doctor?	12	4.3	268	95.7
Have you ever received information about foot care whilst waiting to see the doctor?	10	3.6	270	96.4
Have you ever read any hand-outs on foot care/foot wear?	37	13.2	243	86.8
Would you like a hand-out on how to care for your feet?	268	95.7	12	4.3

DM, diabetes mellitus.

**TABLE 3:** Practice of diabetic foot care.

	Yes		No	
		
Practice questions	*n*	%	*n*	%
Can you reach your feet?	193	68.9	87	31.1
Do you examine your feet?	150	65.2	130	34.8
Do you wash your feet every day?	277	98.9	3	1.1
Do you dry between your toes?	260	92.9	20	7.1
Do you use cream on your feet?	98	35.0	182	65.0
Do you use cream between your toes?	84	30.0	196	70.0
Do you use medicated foot products?	28	10.0	252	90.0
Do you file your toenails?	200	71.4	80	28.6
Do you trim your toenails?	265	94.6	15	5.4
Do you walk barefoot?	132	47.1	148	52.9
Do you inspect your shoes prior to wearing them?	245	87.5	35	12.5
Do you soak your feet?	158	56.4	122	43.6
If Yes above, do you check the water temperature before soaking your feet?	139	88.0	19	12.0
Do you use a hot-water bottle on your feet?	31	11.1	249	88.9
Do you sit with your legs crossed?	93	33.2	187	66.8
Do you smoke?	26	9.3	254	90.7

Most patients believed that DM would eventually lead to DFD, that smoking contributes to reduced blood flow to the limbs, and that dietary measures are important in controlling DM. They also believed that they could live a normal life notwithstanding T2DM. Most participants said that foot examination should be their own responsibility. More than 90% of participants had not received any previous form of DFC education from any source.

Sixty-five per cent of participants examined their feet every day, and 22.2% examined their feet only when they had a problem. Ninety-four per cent of participants who did not cut their own toenails had it done by a family member. Of the 265 participants who trimmed their toenails, 79.3% trimmed their nails along the edges and 20.7% trimmed them straight. Participants wore more than one type of shoe, with sandals and flip-flops being used by 83.2% of participants. Approximately 88% of participants wore socks regularly.

Table 4 shows descriptive statistics of knowledge, attitudes, practices and previous foot education scores.

**TABLE 4:** Descriptive statistics of knowledge score, attitude score, practice score and previous foot education score.

Scores	*N*	Mean	s.d.	CI
Knowledge score	280	4.45	2.201	4.2–4.7
Attitude score	280	2.67	0.556	2.6–2.7
Practice score	280	11.09	2.233	10.8–11.5
Previous foot education score	280	0.63	1.203	0.5–0.8

s.d., standard deviation; CI, 95% confidence interval; *N*, number.

Knowledge scores ranged from 0 – 8, attitude scores from 0 – 3, practice scores from 0 – 19, and previous foot education from 0 – 7. High scores represent best responses. The average attitude score was 2.67 with a low s.d. Previous foot education scores had the lowest mean.

**FIGURE 3:**
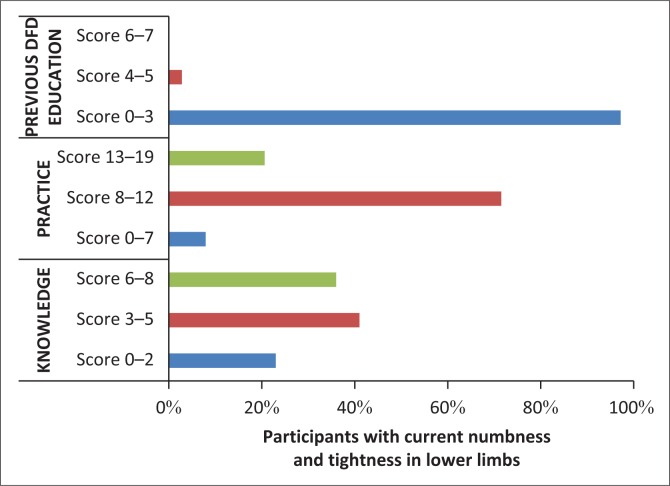
Scores for knowledge, practice and previous education on DFD, for participants with current numbness and tightness in lower limbs.

Most participants with altered sensation in their lower limbs had not received previous DFD education and they had mediocre scores and average scores for knowledge and practice of DFC respectively (Figure 3).

The scores for participants with no altered sensation in their lower limbs (23.6%, *n* = 66) were significantly the same.

Table 5 presents the levels of significance of demographic variables and clinical profiles of participants compared with knowledge, attitude, practice and previous foot education regarding DFC.

**TABLE 5:** Impact of demographic factors and clinical profile on patients’ knowledge and practice of diabetic foot care.

	Knowledge		Attitude		Practice		Previous foot care education	
				
Variable	*r*	*p*	*r*	*p*	*r*	*p*	*r*	*p*
Gender	0.070	0.123	0.168	0.002	0.062	0.152	0.069	0.126
18–34 years old	0.060	0.159	0.051	0.199	0.092	0.063	0.062	0.151
35–49 years old	0.047	0.216	0.097	0.052	0.065	0.138	0.068	0.128
50–64 years old	-0.002	0.486	-0.054	0.183	-0.018	0.384	0.039	0.258
>65 years old	-0.045	0.226	-0.025	0.339	-0.048	0.212	-0.106	0.038
Education	-0.090	0.067	0.024	0.347	0.065	0.140	0.171	0.002
DM for <5 years	-0.090	0.660	0.072	0.116	-0.310	0.304	-0.068	0.128
DM for ≥5 years	0.093	0.610	0.077	0.101	0.270	0.326	0.077	0.098

*r*, Pearson coefficient; *p*, *p*-value; DM, diabetes mellitus.

There was a positive relationship between gender and attitude, with a positive Pearson coefficient (*r*) of 0.168 and a *p*-value that was significant (*p* < 0.05). Men had a significantly higher attitude score than that of women. There was also a positive relationship between education level and previous foot care education (*p* < 0.05), with patients having a higher level of education achieving a better score. There was a significant negative correlation between participants aged <65 and the score for previous foot care education (*p* < 0.05), implying that people <65 are more likely to report a higher score for previous foot care education than people ≥65.

## Discussion

Despite the systematic randomised sampling method used, the genders were not equally represented, with 201 female v. 79 male participants in the study. Although the prevalence of T2DM is unequal amongst the genders worldwide, with more men having T2DM than women, studies have shown that there are more women with T2DM in SA and that more women attend outpatient clinics than do men.^2,32,33,34,35^ The mean age of 59 years is consistent with global figures of people with DM.^2^

A large percentage of participants had a high body mass index (BMI), with a mean BMI of 33.4. This is a concern as obesity is a major risk factor for DM complications. Despite 87% of participants claiming to be on a diabetic diet, only 29% claimed to be on a low-fat no-sugar regime. A large percentage of participants acknowledged that diet was important in the control of their DM but only a minority of participants monitored their weight, which is consistent with studies elsewhere.^36^ Results of a recent BMI study and related mortality of patients with T2DM have indicated a J-shaped relationship between the two concepts, with an exponential increase in mortality demonstrated amongst T2DM patients who have higher BMIs.^36^

There is strong evidence that lifestyle modifications such as all types of physical activity, losing weight, ceasing smoking and following nutritional recommendations provide benefits in the prevention of hypertension, dyslipidaemia, diabetes and other cardiovascular diseases associated with T2DM, and can reduce DM itself.^37,38^ Of concern in our study is that the majority of participants were already affected by cardiovascular diseases. It was encouraging, however, that only 9% of participants smoked cigarettes, which may be related to the participants’ racial composition. A study in South Africa that analysed the correlation between race and smoking status amongst adult South Africans concluded that smoking habits may be related to race.^39^ In SA, black people have the lowest smoking prevalence, and this prevalence is decreasing.^40,41^ Given that blacks represent about 75% of the total population in SA and almost 90% of our sample size, this finding may explain the relatively low smoking rates found in the present study. In DM, smoking accounts for more than half of the risks contributing to the development of peripheral artery disease and DFD.^42,43,44^ Participants had a fair level of awareness about the effect of smoking on the reduction of blood flow to the lower limbs. Generally, knowledge influences behaviour, which may be one of the reasons why a large proportion of participants were non-smokers.^45,46^ Improving knowledge by using smoking cessation interventions amongst T2DM patients may be a good public health strategy in decreasing the risks of DFD.^40,41,47^

Although the knowledge score had a high mean, there was great variance demonstrated by the high standard deviation. Participants had poor knowledge of the most significant factors for all types of DFD, namely the presence of foot infection/ulcer, peripheral neuropathy and peripheral vascular disease, which was consistent with other international studies.^44,48^ However, these results represent a slight improvement over previous findings of a South African study in which diabetic patients of black descent had poor knowledge of self-management of their disease.^4,49^ In our study, those at high risk for foot disease had overall average knowledge scores as well as foot care practices, as shown in Figure 3. Poor knowledge may be related to lack of provision of diabetic foot care education as well as the short period from the time that the diagnosis of DM was made, but this correlation was not demonstrated in the present study.^50,51^ A large retrospective cohort study emphasised the importance of screening for symptoms of foot disease in order to implement measures for the secondary prevention of DFD. All diabetic patients should be thoroughly screened for foot disease to identify at-risk feet.^44,48^ Confirmed peripheral neuropathy was found in only 3% of participants. Screening questions revealed that the majority of respondents had numbness and tightness in their lower limbs, a major symptom leading to DFD which requires that stringent management of associated risk factors be implemented to prevent progression of the disease.^52^ Ongoing integrated motivation and education leading to behaviour change should be given to diabetic patients at the first onset of symptoms of foot disease, as the risk of developing DFD is significantly high.^52^ Regular screening of T2DM patients for sensory and vascular foot changes and patient education programmes on DFD including advice on preventative actions, should be highlighted and reinforced in health institutions.^11,44,48,51,53,54^ Daily foot inspection by patients can prevent DFD and its fatal complications. The importance of good knowledge and practice should be stressed and reinforced in T2DM patients who have established peripheral neuropathy so as to reduce the number of diabetic amputations and improve survival. Those with positive features on a screening tool should have much tighter glycaemic control and be subject to more intensive foot care education programmes, with regular scheduled follow-up visits.^7,51^ Podiatrist services should be widely available at PHC level.^55,56^

In our study, foot care practice was poor, with a large variance. To avoid trauma to their lower limbs, T2DM patients with poor vision should let another party examine their feet, and their toenails should be trimmed using the correct technique, which is straight across.^52^ Footwear usage was inappropriate, as many claimed to wear open footwear which would make them more prone to trauma and infection.^22,23,51,52^ Studies have shown that there are reduced rates of DFD in patients when several intervention programmes, including footwear, are implemented.^22,51^ Counselling on the use of appropriate footwear is easy to implement in clinical practice, with the only problem being non-compliance with the prescribed footwear owing to poor socio-economic circumstances or personal preference.

The diabetic foot programme encompasses screening, examination, diagnostic tests, footwear recommendation, referrals, follow-ups and patient education. Diabetic foot education is an essential tool in diabetic foot programmes.^51^ Unfortunately, a considerable number of T2DM patients were not offered adequate self-care foot education, despite the presence of threatening risk factors for lower limb complications. The overall score for previous foot education in our study was poor, which was similar to the result obtained from a study conducted in a PHC setting in Nigeria.^21^ Applied structured educational interventions on diabetic foot education are highly effective in improving knowledge and practices of T2DM patients about DFC.^7,26,50^ The results of a randomised controlled study on foot care education with group or individual counselling demonstrated a significant increase in diabetic patients’ self-efficacy in caring for their feet. Neither of the two training methods (group or individual) is superior to the other in preventing DFD. Both methods show a similar positive effect in preventing DFD.^26^ Group counselling could be a realistic intervention for the South African setting which is often plagued with limited resources. However, in two other studies, a greater improvement in knowledge and practices was shown with individual counselling than with group education, especially in people with long-duration DM as it was postulated that they had impaired cognition owing to their long-standing illness.^50,51^ Group sessions are cost- and time-effective, whilst individual sessions provide better interaction but require more human resources.^26,50^ A structured programme of regular group education and feedback can easily be implemented in public sector settings with minimal use of resources. Peer education is another form of intervention that can easily be implemented at PHC or hospital level, and will improve awareness, attitude and practices of people with T2DM and hopefully improve clinical outcomes.

Men had an overall higher score for attitudes and practice, with a significant difference shown in the attitude score. Some studies found that men demonstrated better abilities than women with, however, some exceptions for women regarding certain specific self-care tasks.^57^ The positive relationship between education level and previous foot care education means that better educated individuals are more likely to demonstrate high scores for previous foot care education as they might be able to access information from various sources such as the internet or a library.

In the present study, we were able to identify participants with at-risk feet. However, we could not determine whether this was related to poor previous foot education of the participants.

## Conclusion and recommendations

DFD causes deterioration in quality of life and affects the quality of care for diabetic patients. It poses a serious medical, social and economic challenge for the healthcare system. Poor knowledge combined with poor self-care practices compromises holistic patient care. The cost of managing DFD encompasses the care of foot ulcers, peripheral vascular diseases, peripheral neuropathy, foot infections and deformities. Many of these foot problems could be reduced if primary and secondary prevention were prioritised in routine clinical care. The patient with T2DM plays a crucial role in preventing foot disease. However, the healthcare system should empower diabetic patients with knowledge, skills and own foot care practices.

The level of awareness of DFD was found to be suboptimal in our study. Furthermore, the clinical management of DFD was incidentally found to be poor. Better screening using a simple tool and a secondary prevention programme, as well as patient education, should be provided at chronic outpatients departments of the study’s research site to minimise the burden of DFD. Prevention, however, requires adequate awareness of the problem. The study results highlighted areas in which preventative efforts to improve knowledge, attitudes and practices could be made. Effective strategies must focus on an aggressive approach to risk factor modifications, footwear inspection and advice, identification of at-risk feet, and diabetic foot care education. Ensuring that patients adhere to non-pharmacological and pharmacological measures is also crucial in ensuring success. An integrated patient-centered approach has been shown to provide adequate incentive to ensure patient adherence management plans.^58^

Specific recommendations to improve the quality of life of T2DM patients attending outpatient clinics include:

- introduction of regular screening for DFD when counselling T2DM patients
- providing group counselling on DFC regularly, using a diabetic educator in resource-limited settings and individual counselling in other settings
- facilitating peer support groups at health facilities so that patients can share knowledge of DFD and DFC
- collaborating with other healthcare providers such as podiatrists to provide comprehensive DFC
- establishing dedicated foot clinics
- empowering patients to request a foot examination yearly from their healthcare provider and that the findings be documented in clinical notes
- regularly evaluate the quality of life of patients with DFD and the impact on daily living activities
- implementing regular clinical audits to ensure standards of care for diabetic patients
- scheduled follow-ups and appropriate referrals
- assessing and grading those diabetic patients with at-risk feet, and active management of DFD features to reduce further incidence of DFD
- appropriate footwear recommendations, taking account of patients’ socio-economic circumstances.
